# *cis*-(Dimethyl sulfoxide-κ*O*)[*N*′-(3-eth­oxy-2-oxidobenzyl­idene-κ*O*)-2-hy­droxy­benzohydrazidato-κ^2^ *N*′,*O*]dioxido­molybdenum(VI)

**DOI:** 10.1107/S1600536811020290

**Published:** 2011-06-04

**Authors:** Ngui Khiong Ngan, Kong Mun Lo, Chee Seng Richard Wong

**Affiliations:** a Department of Chemistry, University of Malaya, 50603 Kuala Lumpur, Malaysia

## Abstract

The coordination geometry at the Mo^VI^ atom in the title compound, [Mo(C_16_H_14_N_2_O_4_)O_2_(C_2_H_6_OS)], is distorted octa­hedral. The phenolate O, imino N, oxide O from the enolized carbonyl group and one of the terminal O atoms form the equatorial plane; the axial positions are occupied by the other terminal O atom of the dioxidomolybdenum group and the donor O atom of DMSO. The O=Mo=O angle is 105.31 (6)°. An intra­molecular O—H⋯N hydrogen bond and weak inter­molecular C—H⋯O hydrogen bonds are present in the structure.

## Related literature

For related Schiff base complexes of molybdenum, see: Rajan & Chakravorty (1981 ▶). For Mo=O bond lengths in *cis*-di­oxidomolybdenum(VI) complexes, see: Dinda *et al.* (2006 ▶); Rao *et al.* (1999 ▶); Syamal & Maurya (1986 ▶).
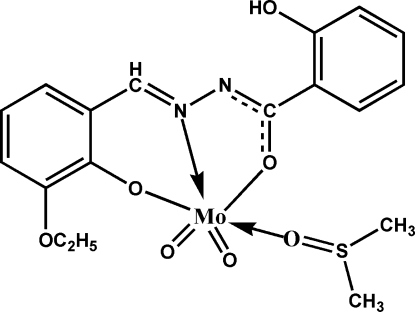


## Experimental

### 

#### Crystal data

[Mo(C_16_H_14_N_2_O_4_)O_2_(C_2_H_6_OS)]
*M* *_r_* = 504
Monoclinic, 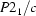

*a* = 7.7527 (1) Å
*b* = 20.6173 (4) Å
*c* = 12.6506 (2) Å
β = 100.931 (1)°
*V* = 1985.38 (6) Å^3^
*Z* = 4
Mo *K*α radiation
μ = 0.81 mm^−1^
*T* = 100 K
0.37 × 0.30 × 0.30 mm

#### Data collection

Bruker APEXII CCD area-detector diffractometer
Absorption correction: multi-scan (*SADABS*; Sheldrick, 1996 ▶) *T*_min_ = 0.752, *T*_max_ = 0.788
18375 measured reflections
4556 independent reflections
4485 reflections with *I* > 2σ(*I*)
*R*_int_ = 0.017

#### Refinement

*R*[*F* ^2^ > 2σ(*F* ^2^)] = 0.024
*wR*(*F* ^2^) = 0.120
*S* = 1.15
4556 reflections
266 parameters
H-atom parameters constrained
Δρ_max_ = 0.47 e Å^−3^
Δρ_min_ = −1.68 e Å^−3^

### 

Data collection: *APEX2* (Bruker, 2008 ▶); cell refinement: *SAINT* (Bruker, 2008 ▶); data reduction: *SAINT*; program(s) used to solve structure: *SHELXS97* (Sheldrick, 2008 ▶); program(s) used to refine structure: *SHELXL97* (Sheldrick, 2008 ▶); molecular graphics: *X-SEED* (Barbour, 2001 ▶); software used to prepare material for publication: *publCIF* (Westrip, 2010 ▶).

## Supplementary Material

Crystal structure: contains datablock(s) I, global. DOI: 10.1107/S1600536811020290/xu5215sup1.cif

Structure factors: contains datablock(s) I. DOI: 10.1107/S1600536811020290/xu5215Isup2.hkl

Additional supplementary materials:  crystallographic information; 3D view; checkCIF report

## Figures and Tables

**Table 1:** Selected bond lengths (Å)

Mo1—N1	2.2343 (13)
Mo1—O1	1.9197 (11)
Mo1—O2	1.7132 (11)
Mo1—O3	1.7055 (12)
Mo1—O4	2.0297 (11)
Mo1—O6	2.2928 (11)

**Table 2:** Hydrogen-bond geometry (Å, °)

*D*—H⋯*A*	*D*—H	H⋯*A*	*D*⋯*A*	*D*—H⋯*A*
O7—H7⋯N2	0.82	1.87	2.5859 (19)	145
C9—H9⋯O3^i^	0.93	2.54	3.217 (2)	130
C18—H18*C*⋯O3^ii^	0.96	2.56	3.438 (2)	152

Symmetry codes: (i) 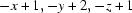; (ii) 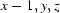.

**Fig. 1.**
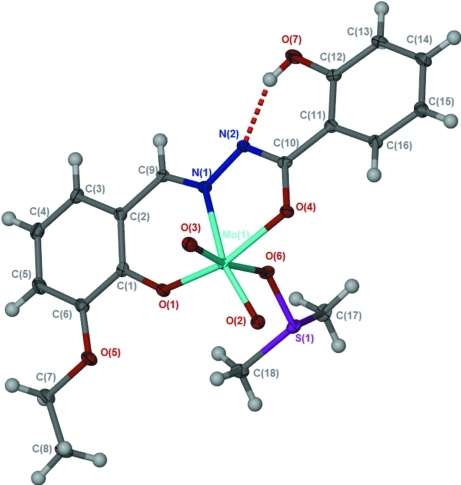
The molecular structure of cisdioxo(3-ethoxysalicylaldehyde- 2-hydroxybenzoylhydrazonato-κ3-O,N,O)dimethyl sulfoxide κ-Omolybdenum(VI)showing 50% probability displacement ellipsoids and the atom numbering. Hydrogen atoms are drawn as spheres of arbitrary radius.

## supplementary crystallographic information

### Comment

The coordination chemistry of molybdenum has taken cognizance of by the scientific community in the last 20 years is because of its ability to achieve multiple common oxidation states ranging from +4 to +6 as well as to form stable complexes with Schiff base ligands (Rajan & Chakravorty, 1981). The title compound represents one of the stable molybdenum complex containing a tridentate *O,N,O'* Schiff base ligand. This molybdenum complex consists of a discrete mononuclear unit (Scheme 1). The overall geometry at molybdenum is a six coordinate octahedron with the bonds formed by the dibasic tridentate ligands together with the two terminal oxygen atoms and the donor oxygen atom of DMSO. The relatively long bond length between Mo and O6 from the DMSO molecule [2.293 (1) Å] shows that the coordination site is labile. The Mo = O bond distances are 1.706 (1) and 1.713 (1) Å, which fall in the expected range for most of the *cis*-dioxomolybdenum(VI) complexes (Dinda, *et al.*, 2006; Syamal & Maurya, 1986 and Rao, *et al.*, 1999).

### Experimental

The Schiff base ligand was prepared by the condensation reaction of salicylic acid hydrazide with 3-ethoxysalicylaldehyde. The title compound was prepared from the equimolar amount of the prepared Schiff base (0.30 g, 1.0 mmol) and bis(acetylacetonato)dioxomolybdenum(VI), [MoO_2_(acac)_2_] (0.328 g, 1.0 mmol) in refluxing ethanol (100 ml). The solution was then added with a few drops of DMSO and refluxed for another 1 h. The solution was left for recrystallizaton at room temperature during which orange colour crystals were obtained.

### Refinement

Hydrogen atoms were placed at calculated positions (C–H 0.93 to 0.97 Å) and were treated as riding on their parent carbon atoms, with *U*_iso_(H) = 1.2–1.5 times *U*_eq_(C). The hydroxy-H was refined with a restraint of 0.82 ± 0.01 Å.

### Crystal data

**Table:**

[Mo(C_16_H_14_N_2_O_4_)O_2_(C_2_H_6_OS)]	*F*(000) = 1024
*M**_r_* = 504	*D*_x_ = 1.677 Mg m^−^^3^
Monoclinic, *P*2_1_/*c*	Mo *K*α radiation, λ = 0.71073 Å
Hall symbol: -P 2ybc	Cell parameters from 9911 reflections
*a* = 7.7527 (1) Å	θ = 2.6–28.3°
*b* = 20.6173 (4) Å	µ = 0.81 mm^−^^1^
*c* = 12.6506 (2) Å	*T* = 100 K
β = 100.931 (1)°	Block, orange
*V* = 1985.38 (6) Å^3^	0.37 × 0.30 × 0.30 mm
*Z* = 4	

### Data collection

**Table:**

Bruker APEXII CCD area-detector diffractometer	4556 independent reflections
Radiation source: fine-focus sealed tube	4485 reflections with *I* > 2σ(*I*)
graphite	*R*_int_ = 0.017
ω scans	θ_max_ = 27.5°, θ_min_ = 2.0°
Absorption correction: multi-scan (*SADABS*; Sheldrick, 1996)	*h* = −10→10
*T*_min_ = 0.752, *T*_max_ = 0.788	*k* = −26→26
18375 measured reflections	*l* = −16→16

### Refinement

**Table:**

Refinement on *F*^2^	Primary atom site location: structure-invariant direct methods
Least-squares matrix: full	Secondary atom site location: difference Fourier map
*R*[*F*^2^ > 2σ(*F*^2^)] = 0.024	Hydrogen site location: inferred from neighbouring sites
*wR*(*F*^2^) = 0.120	H-atom parameters constrained
*S* = 1.15	*w* = 1/[σ^2^(*F*_o_^2^) + (0.1*P*)^2^] where *P* = (*F*_o_^2^ + 2*F*_c_^2^)/3
4556 reflections	(Δ/σ)_max_ = 0.002
266 parameters	Δρ_max_ = 0.47 e Å^−^^3^
0 restraints	Δρ_min_ = −1.68 e Å^−^^3^

### Special details

**Table:**

Geometry. All e.s.d.'s (except the e.s.d. in the dihedral angle between two l.s. planes) are estimated using the full covariance matrix. The cell e.s.d.'s are taken into account individually in the estimation of e.s.d.'s in distances, angles and torsion angles; correlations between e.s.d.'s in cell parameters are only used when they are defined by crystal symmetry. An approximate (isotropic) treatment of cell e.s.d.'s is used for estimating e.s.d.'s involving l.s. planes.
Refinement. Refinement of *F*^2^ against ALL reflections. The weighted *R*-factor *wR* and goodness of fit *S* are based on *F*^2^, conventional *R*-factors *R* are based on *F*, with *F* set to zero for negative *F*^2^. The threshold expression of *F*^2^ > σ(*F*^2^) is used only for calculating *R*-factors(gt) *etc*. and is not relevant to the choice of reflections for refinement. *R*-factors based on *F*^2^ are statistically about twice as large as those based on *F*, and *R*- factors based on ALL data will be even larger.

### Fractional atomic coordinates and isotropic or equivalent isotropic displacement parameters (Å^2^)

**Table:**

	*x*	*y*	*z*	*U*_iso_*/*U*_eq_	
Mo1	0.347736 (16)	0.942369 (6)	0.250939 (9)	0.01077 (11)	
O4	0.40367 (15)	1.02857 (5)	0.18762 (9)	0.0140 (2)	
O6	0.06983 (15)	0.98254 (5)	0.19413 (9)	0.0149 (2)	
O5	0.09177 (17)	0.79029 (6)	0.41705 (10)	0.0189 (3)	
O2	0.30947 (16)	0.89967 (5)	0.13261 (9)	0.0159 (2)	
O1	0.22888 (15)	0.89225 (5)	0.34315 (9)	0.0145 (2)	
C1	0.1871 (2)	0.89761 (7)	0.44109 (12)	0.0132 (3)	
C2	0.2087 (2)	0.95550 (8)	0.50111 (13)	0.0137 (3)	
C9	0.2673 (2)	1.01532 (7)	0.45991 (13)	0.0131 (3)	
H9	0.2699	1.0524	0.5019	0.016*	
N1	0.31605 (17)	1.02025 (6)	0.36858 (11)	0.0129 (3)	
N2	0.35536 (18)	1.08258 (7)	0.33859 (11)	0.0144 (3)	
C10	0.3984 (2)	1.08222 (7)	0.24361 (13)	0.0135 (3)	
C11	0.4365 (2)	1.14361 (7)	0.19552 (12)	0.0148 (3)	
C16	0.4873 (2)	1.14481 (8)	0.09462 (13)	0.0149 (3)	
H16	0.5033	1.1058	0.0608	0.018*	
C15	0.5142 (2)	1.20234 (8)	0.04451 (13)	0.0179 (3)	
H15	0.5455	1.2021	−0.0229	0.021*	
C14	0.4938 (2)	1.26079 (8)	0.09593 (15)	0.0205 (3)	
H14	0.5119	1.2997	0.0625	0.025*	
C13	0.4469 (3)	1.26160 (8)	0.19596 (14)	0.0233 (4)	
H13	0.4356	1.3009	0.2300	0.028*	
C12	0.4164 (2)	1.20308 (8)	0.24641 (13)	0.0185 (3)	
O7	0.3642 (2)	1.20795 (6)	0.34234 (11)	0.0278 (3)	
H7	0.3432	1.1717	0.3633	0.042*	
C3	0.1605 (2)	0.95732 (9)	0.60289 (13)	0.0166 (3)	
H3	0.1750	0.9954	0.6429	0.020*	
C4	0.0923 (2)	0.90354 (8)	0.64354 (13)	0.0190 (3)	
H4	0.0611	0.9053	0.7109	0.023*	
C5	0.0698 (2)	0.84628 (8)	0.58431 (14)	0.0196 (3)	
H5	0.0246	0.8099	0.6129	0.024*	
C6	0.1139 (2)	0.84283 (8)	0.48301 (13)	0.0157 (3)	
C7	0.0258 (2)	0.73202 (8)	0.45849 (14)	0.0189 (3)	
H7B	−0.0908	0.7392	0.4735	0.023*	
H7A	0.1028	0.7184	0.5244	0.023*	
C8	0.0207 (2)	0.68153 (8)	0.37203 (15)	0.0204 (3)	
H8C	−0.0502	0.6969	0.3062	0.031*	
H8A	−0.0289	0.6422	0.3938	0.031*	
H8B	0.1378	0.6733	0.3609	0.031*	
S1	−0.05345 (6)	0.948802 (19)	0.10195 (3)	0.01434 (13)	
C17	−0.2453 (2)	0.99843 (9)	0.08388 (14)	0.0188 (3)	
H17A	−0.3366	0.9787	0.0319	0.028*	
H17C	−0.2186	1.0405	0.0588	0.028*	
H17B	−0.2841	1.0028	0.1512	0.028*	
C18	−0.1346 (2)	0.87857 (9)	0.15964 (14)	0.0215 (3)	
H18A	−0.0400	0.8486	0.1819	0.032*	
H18B	−0.2239	0.8583	0.1071	0.032*	
H18C	−0.1834	0.8912	0.2209	0.032*	
O3	0.55972 (16)	0.92526 (6)	0.30915 (10)	0.0170 (2)	

### Atomic displacement parameters (Å^2^)

**Table:**

	*U*^11^	*U*^22^	*U*^33^	*U*^12^	*U*^13^	*U*^23^
Mo1	0.01172 (15)	0.00986 (15)	0.01120 (15)	0.00052 (3)	0.00331 (9)	−0.00040 (4)
O4	0.0170 (5)	0.0116 (5)	0.0144 (5)	−0.0012 (4)	0.0056 (4)	−0.0013 (4)
O6	0.0131 (5)	0.0157 (5)	0.0150 (5)	0.0005 (4)	0.0005 (4)	−0.0031 (4)
O5	0.0266 (6)	0.0130 (6)	0.0197 (6)	−0.0034 (4)	0.0109 (5)	−0.0007 (4)
O2	0.0194 (6)	0.0147 (6)	0.0143 (5)	0.0012 (4)	0.0046 (4)	−0.0010 (4)
O1	0.0182 (6)	0.0116 (5)	0.0149 (5)	−0.0012 (4)	0.0063 (4)	−0.0003 (4)
C1	0.0125 (7)	0.0155 (7)	0.0119 (7)	0.0027 (5)	0.0030 (5)	0.0007 (5)
C2	0.0139 (7)	0.0139 (7)	0.0136 (7)	−0.0005 (6)	0.0031 (6)	0.0008 (6)
C9	0.0147 (7)	0.0112 (7)	0.0131 (7)	0.0002 (5)	0.0019 (6)	−0.0003 (5)
N1	0.0111 (6)	0.0121 (6)	0.0150 (6)	−0.0006 (5)	0.0010 (5)	−0.0010 (5)
N2	0.0172 (7)	0.0112 (7)	0.0143 (6)	−0.0024 (5)	0.0018 (5)	−0.0002 (5)
C10	0.0114 (7)	0.0129 (8)	0.0157 (7)	0.0001 (6)	0.0014 (5)	−0.0021 (6)
C11	0.0141 (7)	0.0134 (7)	0.0157 (7)	−0.0009 (5)	−0.0004 (6)	0.0005 (6)
C16	0.0124 (7)	0.0150 (7)	0.0168 (7)	−0.0017 (5)	0.0014 (6)	0.0012 (6)
C15	0.0158 (8)	0.0179 (8)	0.0194 (7)	−0.0024 (6)	0.0018 (6)	0.0033 (6)
C14	0.0210 (8)	0.0155 (8)	0.0246 (8)	−0.0075 (6)	0.0030 (6)	0.0022 (6)
C13	0.0333 (10)	0.0130 (8)	0.0235 (9)	−0.0046 (7)	0.0047 (7)	−0.0019 (6)
C12	0.0232 (9)	0.0154 (8)	0.0160 (7)	−0.0037 (6)	0.0014 (6)	−0.0023 (6)
O7	0.0516 (9)	0.0146 (6)	0.0201 (6)	−0.0076 (5)	0.0139 (6)	−0.0036 (5)
C3	0.0188 (8)	0.0181 (7)	0.0126 (7)	0.0007 (6)	0.0024 (6)	−0.0002 (6)
C4	0.0228 (8)	0.0221 (8)	0.0132 (7)	0.0018 (6)	0.0062 (6)	0.0003 (6)
C5	0.0241 (9)	0.0175 (8)	0.0193 (8)	−0.0017 (6)	0.0094 (7)	0.0029 (6)
C6	0.0172 (7)	0.0130 (7)	0.0176 (7)	0.0016 (6)	0.0052 (6)	0.0007 (6)
C7	0.0223 (8)	0.0145 (7)	0.0216 (8)	−0.0026 (6)	0.0082 (6)	0.0023 (6)
C8	0.0208 (8)	0.0139 (8)	0.0269 (9)	−0.0036 (6)	0.0058 (7)	−0.0015 (6)
S1	0.0125 (2)	0.0178 (2)	0.0127 (2)	0.00100 (12)	0.00257 (17)	−0.00274 (13)
C17	0.0112 (7)	0.0249 (8)	0.0193 (8)	0.0036 (6)	0.0004 (6)	0.0004 (6)
C18	0.0202 (8)	0.0185 (8)	0.0249 (8)	−0.0069 (6)	0.0021 (6)	−0.0019 (6)
O3	0.0161 (6)	0.0142 (5)	0.0203 (6)	0.0015 (4)	0.0024 (5)	0.0005 (5)

### Geometric parameters (Å, °)

**Table:**

Mo1—N1	2.2343 (13)	C14—C13	1.381 (2)
Mo1—O1	1.9197 (11)	C14—H14	0.9300
Mo1—O2	1.7132 (11)	C13—C12	1.406 (2)
Mo1—O3	1.7055 (12)	C13—H13	0.9300
Mo1—O4	2.0297 (11)	C12—O7	1.354 (2)
Mo1—O6	2.2928 (11)	O7—H7	0.8200
O4—C10	1.3184 (19)	C3—C4	1.370 (2)
O6—S1	1.5278 (12)	C3—H3	0.9300
O5—C6	1.358 (2)	C4—C5	1.391 (2)
O5—C7	1.4425 (19)	C4—H4	0.9300
O1—C1	1.3431 (17)	C5—C6	1.389 (2)
C1—C2	1.407 (2)	C5—H5	0.9300
C1—C6	1.412 (2)	C7—C8	1.505 (2)
C2—C3	1.408 (2)	C7—H7B	0.9700
C2—C9	1.445 (2)	C7—H7A	0.9700
C9—N1	1.286 (2)	C8—H8C	0.9600
C9—H9	0.9300	C8—H8A	0.9600
N1—N2	1.3901 (19)	C8—H8B	0.9600
N2—C10	1.307 (2)	S1—C17	1.7840 (16)
C10—C11	1.459 (2)	S1—C18	1.7886 (18)
C11—C16	1.406 (2)	C17—H17A	0.9600
C11—C12	1.407 (2)	C17—H17C	0.9600
C16—C15	1.379 (2)	C17—H17B	0.9600
C16—H16	0.9300	C18—H18A	0.9600
C15—C14	1.393 (2)	C18—H18B	0.9600
C15—H15	0.9300	C18—H18C	0.9600
			
O3—Mo1—O2	105.31 (6)	C14—C13—C12	120.11 (15)
O3—Mo1—O1	99.24 (5)	C14—C13—H13	119.9
O2—Mo1—O1	103.34 (5)	C12—C13—H13	119.9
O3—Mo1—O4	95.22 (5)	O7—C12—C13	116.61 (15)
O2—Mo1—O4	96.87 (5)	O7—C12—C11	123.54 (15)
O1—Mo1—O4	150.95 (5)	C13—C12—C11	119.83 (15)
O3—Mo1—N1	94.57 (5)	C12—O7—H7	109.5
O2—Mo1—N1	158.21 (5)	C4—C3—C2	120.67 (16)
O1—Mo1—N1	81.72 (5)	C4—C3—H3	119.7
O4—Mo1—N1	72.08 (5)	C2—C3—H3	119.7
O3—Mo1—O6	168.91 (5)	C3—C4—C5	120.18 (15)
O2—Mo1—O6	85.12 (5)	C3—C4—H4	119.9
O1—Mo1—O6	81.61 (5)	C5—C4—H4	119.9
O4—Mo1—O6	79.54 (4)	C6—C5—C4	120.82 (15)
N1—Mo1—O6	74.56 (4)	C6—C5—H5	119.6
C10—O4—Mo1	119.47 (10)	C4—C5—H5	119.6
S1—O6—Mo1	119.30 (6)	O5—C6—C5	125.65 (14)
C6—O5—C7	116.94 (12)	O5—C6—C1	114.85 (13)
C1—O1—Mo1	138.04 (10)	C5—C6—C1	119.50 (14)
O1—C1—C2	122.86 (14)	O5—C7—C8	105.92 (13)
O1—C1—C6	117.71 (13)	O5—C7—H7B	110.6
C2—C1—C6	119.40 (14)	C8—C7—H7B	110.6
C1—C2—C3	119.41 (15)	O5—C7—H7A	110.6
C1—C2—C9	122.85 (14)	C8—C7—H7A	110.6
C3—C2—C9	117.59 (15)	H7B—C7—H7A	108.7
N1—C9—C2	123.88 (14)	C7—C8—H8C	109.5
N1—C9—H9	118.1	C7—C8—H8A	109.5
C2—C9—H9	118.1	H8C—C8—H8A	109.5
C9—N1—N2	115.81 (13)	C7—C8—H8B	109.5
C9—N1—Mo1	129.04 (11)	H8C—C8—H8B	109.5
N2—N1—Mo1	115.16 (10)	H8A—C8—H8B	109.5
C10—N2—N1	110.69 (13)	O6—S1—C17	102.85 (7)
N2—C10—O4	122.56 (14)	O6—S1—C18	105.94 (7)
N2—C10—C11	119.07 (14)	C17—S1—C18	99.63 (9)
O4—C10—C11	118.34 (14)	S1—C17—H17A	109.5
C16—C11—C12	118.33 (14)	S1—C17—H17C	109.5
C16—C11—C10	120.52 (13)	H17A—C17—H17C	109.5
C12—C11—C10	121.10 (14)	S1—C17—H17B	109.5
C15—C16—C11	121.69 (15)	H17A—C17—H17B	109.5
C15—C16—H16	119.2	H17C—C17—H17B	109.5
C11—C16—H16	119.2	S1—C18—H18A	109.5
C16—C15—C14	119.28 (15)	S1—C18—H18B	109.5
C16—C15—H15	120.4	H18A—C18—H18B	109.5
C14—C15—H15	120.4	S1—C18—H18C	109.5
C13—C14—C15	120.75 (15)	H18A—C18—H18C	109.5
C13—C14—H14	119.6	H18B—C18—H18C	109.5
C15—C14—H14	119.6		
			
O3—Mo1—O4—C10	−91.49 (12)	Mo1—N1—N2—C10	2.03 (15)
O2—Mo1—O4—C10	162.37 (11)	N1—N2—C10—O4	−0.7 (2)
O1—Mo1—O4—C10	28.29 (17)	N1—N2—C10—C11	177.19 (13)
N1—Mo1—O4—C10	1.64 (11)	Mo1—O4—C10—N2	−1.2 (2)
O6—Mo1—O4—C10	78.65 (11)	Mo1—O4—C10—C11	−179.08 (10)
O3—Mo1—O6—S1	175.6 (2)	N2—C10—C11—C16	178.44 (15)
O2—Mo1—O6—S1	15.13 (8)	O4—C10—C11—C16	−3.6 (2)
O1—Mo1—O6—S1	−89.16 (7)	N2—C10—C11—C12	−4.2 (2)
O4—Mo1—O6—S1	113.05 (8)	O4—C10—C11—C12	173.75 (15)
N1—Mo1—O6—S1	−172.82 (8)	C12—C11—C16—C15	−1.3 (2)
O3—Mo1—O1—C1	79.54 (15)	C10—C11—C16—C15	176.14 (15)
O2—Mo1—O1—C1	−172.19 (14)	C11—C16—C15—C14	1.3 (2)
O4—Mo1—O1—C1	−39.3 (2)	C16—C15—C14—C13	−0.1 (3)
N1—Mo1—O1—C1	−13.78 (14)	C15—C14—C13—C12	−1.0 (3)
O6—Mo1—O1—C1	−89.27 (15)	C14—C13—C12—O7	−177.42 (17)
Mo1—O1—C1—C2	9.6 (2)	C14—C13—C12—C11	1.0 (3)
Mo1—O1—C1—C6	−172.29 (11)	C16—C11—C12—O7	178.43 (16)
O1—C1—C2—C3	179.48 (14)	C10—C11—C12—O7	1.1 (3)
C6—C1—C2—C3	1.4 (2)	C16—C11—C12—C13	0.1 (2)
O1—C1—C2—C9	4.0 (2)	C10—C11—C12—C13	−177.29 (16)
C6—C1—C2—C9	−174.13 (15)	C1—C2—C3—C4	−0.3 (2)
C1—C2—C9—N1	−4.9 (2)	C9—C2—C3—C4	175.46 (16)
C3—C2—C9—N1	179.57 (15)	C2—C3—C4—C5	−0.1 (3)
C2—C9—N1—N2	175.26 (14)	C3—C4—C5—C6	−0.7 (3)
C2—C9—N1—Mo1	−5.0 (2)	C7—O5—C6—C5	−3.7 (2)
O3—Mo1—N1—C9	−87.68 (14)	C7—O5—C6—C1	176.88 (14)
O2—Mo1—N1—C9	116.33 (17)	C4—C5—C6—O5	−177.63 (16)
O1—Mo1—N1—C9	11.01 (14)	C4—C5—C6—C1	1.8 (3)
O4—Mo1—N1—C9	178.30 (15)	O1—C1—C6—O5	−0.8 (2)
O6—Mo1—N1—C9	94.54 (14)	C2—C1—C6—O5	177.36 (14)
O3—Mo1—N1—N2	92.03 (10)	O1—C1—C6—C5	179.66 (14)
O2—Mo1—N1—N2	−63.95 (18)	C2—C1—C6—C5	−2.1 (2)
O1—Mo1—N1—N2	−169.27 (11)	C6—O5—C7—C8	−177.89 (14)
O4—Mo1—N1—N2	−1.99 (9)	Mo1—O6—S1—C17	−177.76 (7)
O6—Mo1—N1—N2	−85.75 (10)	Mo1—O6—S1—C18	78.13 (9)
C9—N1—N2—C10	−178.22 (14)		

### Hydrogen-bond geometry (Å, °)

**Table:**

*D*—H···*A*	*D*—H	H···*A*	*D*···*A*	*D*—H···*A*
O7—H7···N2	0.82	1.87	2.5859 (19)	145
C9—H9···O3^i^	0.93	2.54	3.217 (2)	130
C18—H18C···O3^ii^	0.96	2.56	3.438 (2)	152

Symmetry codes: (i) −*x*+1, −*y*+2, −*z*+1; (ii) *x*−1, *y*, *z*.
